# The Creation of a Systematic Framework to Assess Dog Laws and Their Relationship to Societal Changes in the United Kingdom

**DOI:** 10.3390/ani15050647

**Published:** 2025-02-23

**Authors:** Sarah A. Weir, Lynsey McDevitt, Clare P. Andrews, Sharon E. Kessler

**Affiliations:** Department of Psychology, Faculty of Natural Sciences, University of Stirling, Stirling FK9 4LA, UK; lynseymcdevitt@aol.com (L.M.); clare.andrews@stir.ac.uk (C.P.A.); sharon.kessler@stir.ac.uk (S.E.K.)

**Keywords:** dogs, companion animals, animal welfare, dog control, dangerous dogs, regulation, breeding, sales, legislation, legal comparison

## Abstract

Dogs and humans have shared a complex relationship throughout history, with law serving as an important tool to manage dogs’ integration into human societies. As dogs increasingly become regarded as family members in Western countries, and as similar trends emerge globally, it is vital to understand how legislation balances the interests of stakeholders. Existing studies often focus on localised disputes and fragmented legal areas, limiting understanding of how dog-related laws interact and potentially conflict. We developed a conceptual framework to systematically analyse dog-related legislation, using the United Kingdom as a case study. Identified through a systematic search, laws were evaluated using content analysis based on the benefits provided to stakeholders, the regulated aspects of dog ownership, and whether benefits afforded to stakeholders occur in public or private spaces. We found that the greatest legislative focus was on dog welfare, dangerous dogs, and dog control, with little emphasis on areas like the breeding and sale of dogs. Eighty-two percent of laws that manage dogs in public space predominantly benefit the general public, often disadvantaging dogs and their owners, while 81% of laws that govern dogs within the home favour dogs. Owners consistently face legal obligations, but gain few benefits. These findings highlight misalignments between the law and dogs’ evolving societal roles, potentially contributing to public space conflicts and low compliance. The framework offers a tool for cross-country comparisons and assessing legislation for other species with similarly shifting roles.

## Simple Summary

Dogs and humans share a complex, intertwined history, and as a result law has served as a crucial tool to manage coexistence in society. As dogs increasingly transition into the role of family members, this change creates new challenges, particularly when it conflicts with those who view dogs as potential nuisances or threats. How governments balance these interests is currently unknown. Using the United Kingdom as a case study, we developed a conceptual framework to systematically compare laws across governments by evaluating the benefits to key stakeholders and the regulated aspects of dog ownership. We found that dog welfare and dangerous dogs dominate legislative focus; however, who benefits depends on the type of law. Laws regulating dogs in public spaces primarily prioritise the interests of the general public, whereas legislation concerning dogs in the home tends to overwhelmingly benefit dogs. Notably, dog owners face extensive legal obligations, yet receive relatively few benefits. These findings have important implications for both dog welfare organisations and policymakers. Dog welfare campaigns may be more successful if they emphasise public benefits. As dogs’ interests are disadvantaged in public space, responsible dog ownership initiatives should distinguish between welfare and control measures, as owners are more likely to comply with regulations that protect their dogs’ interests.

## 1. Introduction

Today and historically, dogs have occupied an important place in the daily lives of people. As a result, governments have used the law to manage dogs since the beginnings of modern legal systems [1,2,3]. Since their domestication, estimated to be between 15,000 to 40,000 years ago [4], dogs have occupied a wide variety of roles in human societies, evolving to meet the changing needs of people. This includes functional roles such as hunting [5,6], transport [7,8,9], herding, and protection for people and livestock [10,11], as well as service dogs providing physical and psychological support for people [12,13,14]. They have also roamed the streets freely [2,15,16], been kept exclusively as pets, especially by royalty and women [1,17], and been used as status symbols [18,19]. Their changing status, paired with their ability to impact everyone in a society by occupying public space, can result in conflict [20]. As law is an important tool used by governments to resolve social problems, often by defining the limits of acceptable behaviour [21], it has led to laws dictating the daily lives of dogs and their interactions with humans. For example, the law can determine how dogs are cared for, how they should behave, to what extent they can enter public spaces, and whether they are the property of humans [22,23,24]. These laws have been made over time, shaped by the underlying beliefs and fears of society [25]. Therefore, a country’s dog laws likely reflect a country’s attitudes about dogs and the roles they hold. This study aimed to create a framework to investigate a country’s laws relating to dogs, whose interests they serve, and how the priorities of different groups are balanced.

Legislation, law passed by legislative bodies, can illuminate how a society perceives dogs. Legislation is an active process with defined goals that are created and built on over time [26]. Therefore, the history we share with dogs is important in shaping legislation, and thus the lives of people and dogs. Legislation played a pivotal role in shaping the current form of Western dog ownership, described by Fox and Gee [27] as intensive pet-keeping. For the purposes of this paper, we use the term ‘owner’ to refer to individuals who live in a household with companion dogs, because this is the term most often used in law and that continues to be used by Western governments. However, we acknowledge that this terminology is increasingly challenged in some academic disciplines, where alternative terms such as ‘guardian’ are preferred [28].

Rooted in the creation of Western modern cities in the 18th and 19th centuries, intensive pet-keeping involves owners integrating dogs into the home while being subject to greater restrictions in public spaces. Initially driven by beliefs about dogs’ associations with rabies, the ‘dangerous classes’, and public disorder, dogs began to be increasingly controlled while in public [29,30]. Western governments enacted legislation permitting the removal and culling of stray dogs, required owned dogs to wear muzzles and be kept on leads in public, and introduced a dog tax [2,31,32]. During the same period, pet-keeping became accessible to the growing middle class, making the practice of keeping dogs solely for companionship (and status) socially acceptable [33]. As the number of working and stray dogs on the streets declined, and pet dogs kept for companionship became more common, the home was perceived as the proper place for dogs [29]. It was also during this period that there was a growing acceptance that animals could suffer, leading to anti-cruelty legislation that aimed to protect animals from their owners [34]. Dogs were perceived by animal protectionists to be among the animals that deserved the most sympathy because of their strong bonds with humans and the belief that they had an increased capacity to feel compared to other animals such as livestock, cats, and birds [2]. Dogs were on the one hand feared and hated as dangerous and potential carriers of disease, and on the other loved and afforded protection as pets in the home. These complicated attitudes towards dogs were then reflected in the legislation passed during this time.

These complex and conflicting ideas about dogs contributed to an emerging divide in how dogs were treated when in private versus when in public, with this divide being cemented into Western societies in the 1970s. Despite government efforts to control dogs in public, dogs in Westen countries were free to roam their neighbourhoods, choosing how to spend their day [27]. However, growing fears over the potential of dogs causing injuries, spreading diseases through their faeces, a renewed rabies scare, and creating environmental and neighbourhood nuisances prompted the introduction of increased legislation controlling dogs in public space [16,35,36]. In the same period, dogs became increasingly incorporated into more-than-human families, often considered as a family member, child, or child substitute [37,38,39,40]. This has been hypothesised to be a result of decreased economic and social security, reduced birth rates, the expanded definition of family, and increased loneliness [41,42,43,44,45]. Changing attitudes towards animals, particularly the creation of animal welfare legislation and the rise of animal rights, also likely contributed to the movement of dogs from pets to family [46,47]. Researchers have argued that these changes have resulted in dogs living separate public and private lives [27,48,49,50]. In private, they are family members who are cared for and afforded similar resources, time, and attention as children [27,38]; in public, they are dangerous, dirty, and annoying property that need to be controlled by their owners [24,50,51].

The welfare and control legislation passed during this period has been described as creating a culture of ‘responsible dog ownership’ [27,52]. This term often describes the need for owners to provide for their dog’s welfare and to prevent their dog from acting in a way that could be perceived as dangerous or a nuisance [27,53]. Borthwick [52] found that laws in New South Wales moved the responsibility of managing dogs from governments to owners. ‘Responsible dog ownership’ aims to oblige dog owners to meet their legal responsibilities, especially as more recent laws are difficult to enforce and require self-enforcement [54]. This creates tension for dog owners who are required to navigate the needs of their dogs, family, society, and themselves. Dog owners are potentially not best placed to manage these tensions because they often prioritise their dogs over others, and define responsible behaviour based on what they personally consider to be best [53,55]. Some responsibilities may be too challenging for owners to fulfil, such as being in control of their dog at all times while simultaneously meeting their dog’s welfare needs or navigating complicated bureaucratic requirements [54,56,57].

These increased expectations on owners coupled with dogs’ lives being split into public and private spheres can create conflict in communities. Dog owners wish to extend their familial connections with their dogs into the public sphere, desiring to bring their dogs into parks, shops, cafes, or workplaces [22,38,51]. These owners believe that if dogs are family, they should have the same rights to public space as any other human family. However, those opposed to dogs in public spaces perceive dogs as dirty, dangerous animals belonging to the private sphere and believe that public spaces should prioritise humans, especially children [50,51]. This suggests that conflict arises from a difference in opinion over which groups should be prioritised in public spaces [50,51,58]. Our study builds on previous work on conflicts in specific contexts, like parks [51,58,59,60], cities [22,61], and provinces [48,50], by examining the role the law plays in balancing the needs of different groups on the larger scale of a country level.

Resolving social issues by defining acceptable behaviour in law usually involves providing benefits for one group of stakeholders, sometimes at the expense of others. Who receives these benefits can be a reflection of these groups’ importance to wider society, as well as the groups who hold power [25]. Law has historically been anthropocentric, reflecting the long-standing belief in Western societies that humans are morally superior to animals [1,62]. This view materialised in the categorisation of dogs (and other non-human animals) as chattel property, the same legal category as tables, chairs, and other inanimate objects [34,63,64,65]. This status means that dogs do not have protected legal rights and instead are subject to the property rights granted to their owners [34,66]. These property rights are limited by anti-cruelty legislation that prohibit harm to dogs and welfare laws that require owners to meet their dogs’ needs [34], resulting in dogs being what Shepard [65] describes as property with limited interests. However, as companion animals become more like family, there are increasing calls to recognise their sentience and challenge the human/animal binary in legislation and the courts [64,67,68,69,70]. This includes recognising animals as sentient in animal welfare legislation [69,71], affording them the ability to be recognized as victims of crime, including domestic abuse [72,73], or creating a new legal status such as ‘sentient property’ or ‘living property’ that allows courts to treat animals as sentient without disrupting human ownership [67,74]. Many of these discussions are ongoing and it is unclear as to the degree they have entered different aspects of dog-related legislation. If dogs are perceived differently in public or private spheres, governments may also prioritise them differently based on the sphere they occupy.

Governments are expected to balance these different human and dog interests along with emerging non-human animal interests like wildlife and ecosystems [48,75,76]. However, a piecemeal, sometimes reactive approach to law-making can result in the purpose of one law conflicting with another or mean that the interests of different parties may not be intentionally balanced within specific pieces of legislation. Dog law is created at different levels of government (national, local, and within the UK, devolved powers) which can lead to variation in legislation within a country [54,77]. Furthermore, legislation impacting dogs does not always explicitly target dogs alone, but includes legislation relating to animals in general, with which we have diverse human–animal relationships. While animal welfare laws are often created for all owned animals (including farmed and zoo animals), laws aiming to manage the negative impacts of dogs are created specifically for them, often in response to intense public pressure [34,78]. For example, dangerous dog legislation was created rapidly across many countries in Europe, banning certain breeds perceived to be particularly dangerous, after a series of incidents involving dogs who attacked and killed children [79,80]. In Britain, this legislation was drafted and passed in just two days, and has since been criticised for being ineffective and devaluing dogs and their owners [79,81,82]. When legislation is enacted suddenly in response to pressure from specific groups, such as the media, it may fail to consider the interaction between laws or balancing the interests of different stakeholder groups.

Creating a holistic understanding of how dog-related legislation prioritises different groups is important because stakeholders, particularly dogs and their owners, must navigate multiple laws on a daily basis. Yet, most research investigating dog-related laws is siloed, with detailed understandings of certain areas of law usually coming from different disciplines. Examples include animal welfare law [34,83,84,85], dangerous dog legislation [57,82,86,87,88,89], managing dogs in public spaces [22,48,51,90], managing dogs’ impact on the environment [91,92,93,94,95], providing for dogs in accommodation [96,97] and exploring animal personhood [64,65,73,98]. This siloed nature of study is likely a result of the different aims of legislation, a focus on creating deep understanding of law, the different interests driving research, and dog-related law differing within countries. For example, Andersen et al. [77] compared dog welfare laws across culturally Western countries, but had to select one or two jurisdictions to represent each country as a whole. These challenges have resulted in few papers systematically comparing laws across jurisdictions, limiting the academic coherence or public understanding within and across countries [22,99,100,101,102]. Since the average dog and owner are subjected to multiple laws simultaneously, further research is needed to understand how laws targeting different areas of dog ownership interact to prioritise different groups and how this might change across contexts.

This paper aims to fill these gaps by creating a novel, systematic framework that can compare laws across governments and law areas by determining the benefits legislation affords to stakeholders and identifying the most commonly regulated aspects of dog ownership. We used the United Kingdom (UK) as a case study to create this framework. Dogs are popular in the UK, where 36% of households own at least one dog [103]. However, this popularity varies across the four nations (England, Scotland, Wales, and Northern Ireland), which also vary politically, demographically, and culturally [104,105]. The UK is a unitary system with partly autonomous (known as devolved) nations, which has resulted in four nations that can create animal-related laws, but three legal systems [106]. This complexity has led to studies often focusing on England to represent the UK as a whole [27,77,107], or on Great Britain, excluding Northern Ireland [24,34,79]. The UK’s laws are especially useful to understand because of Britain’s colonial past. British laws have shaped the legislative approaches of other countries, and so may be relevant to other countries [24,65,108,109]. The present study seeks to create, for the first time, a detailed, systematic understanding of dog-related legislation created by national legislative bodies across the UK, with the goal that this framework can in future be applied in other countries, to enable systematic, international comparisons. We demonstrate how this framework can be used to address questions such as what areas of dog ownership are most legislated, the degree to which stakeholder groups are benefited or disadvantaged by law, and if these legislative approaches differ across the nations.

## 2. Materials and Methods

### 2.1. Creation of a Dog Law Database

To create a database of legislation that impacts dogs and their owners, we included primary and secondary legislation that originated in the UK. Primary legislation is law passed by the UK legislative bodies (UK Parliament, Scottish Parliament, Welsh Parliament, and the Northern Ireland Assembly). Secondary legislation are laws created by ministers under authority provided by primary legislation, with the most common form being statutory instruments [110]. These instruments are created by government ministers to make changes to laws, fill in important details, or amend existing Acts without Parliament needing to pass a new law [111,112]. We have included them in our analysis because the volume of statutory instruments has risen, and they are increasingly used to make substantial changes to legislation or make new rules [111,113].

Primary and secondary legislation was identified using a systematic search of legislation.gov.uk, the official database of UK law hosted by The National Archives. Legislation.gov.uk was chosen for the project because it was created to make the law more accessible and understandable to the general public [26]. Therefore, it would best reflect how an average dog owner could access and understand the law. The site holds all primary legislation in force from 1267 and all secondary legislation since 1823, but does not include legislation which was fully repealed before 1991 [110]. It also holds laws from all four nations, unlike alternatives such as LexusNexus+ which we found did not hold older legislation from Northern Ireland. Therefore, it was the best source to gain access to laws in force across the UK, enabling a fair comparison.

To identify all dog-related laws, we conducted an initial systematic search of legislation.gov.uk between March to May 2022. We identified 316 primary and secondary laws using the search terms ‘dog’, ‘animal welfare -farm -health’ (the words health and farm were excluded because these terms are common in agriculture and are rarely used for domestic companion animals), ‘pet’, ‘companion animal’, and ‘animal protection’, with year = blank, Number = blank, and Type = “All UK Legislation (excluding originating from the EU)”. To ensure we captured all important legislation, we supplemented this search with laws identified in public-facing resources from UK dog organisations [114,115,116,117]. Additionally, later during the coding process, laws referenced in already-included legislation were also added if they met the inclusion criteria (detailed below). Finally, we performed a cross-verification of the laws across nations to identify and address any gaps in the dataset. After duplicates were removed, this process resulted in 361 laws.

Our unit of analysis was the numbered parts of the law, mainly known as sections. Although it is noted that the term can vary depending on the legal instrument and jurisdiction, the term section will be used here because it is the most used across the UK. We used sections rather than laws because there was variation in the length of legislation and the breadth of issues legislated. For example, the Dogs (Northern Ireland) Order 1983 creates requirements regarding dog control, dangerous dogs, microchipping, licensing schemes for dog owners, breeding facilities, and guard dog kennels. In other nations, many of these issues are addressed in multiple pieces of separate legislation. There are also some important dog-related sections located within legislation that legislate a wide range of non-dog related issues such as the Anti-social Behaviour, Crime, and Policing Act 2014. Treating the sections independently allowed for more effective comparisons between the nations.

As a unitary system, the UK Parliament can make laws for all nations, although it usually will not if an issue is devolved [118]. As there is legislation that was passed before powers were devolved, some laws are in effect in multiple nations. For example, the Dangerous Dog Act 1991 applies in England, Scotland, and Wales, except for Section 8, which also applies in Northern Ireland [119]. To assess which nation sections applied to, we used the ‘Show geographic extent’ feature on legislation.gov.uk. This feature showed which nations each section applied to. However, we verified this using the information within the legislation when available.

To ensure only legislation that was still in force was included, repealed laws were identified during the coding process using a number of different sources. Sometimes the status of the law was not updated on legislation.gov.uk, especially for statutory instruments. If the law was not updated, we checked the “Changes to legislation” section on the website and took note of all laws revoked or repealed in the laws we were coding. If there was no confirmation of status, we emailed the relevant archival authority or checked LexusNexus+. Repealed laws constituted 28% (1461) of identified sections.

We also excluded legislation originating from the European Union (EU), because our focus was on laws that were shaped by societal forces in the UK. Additionally, data collection took place while the UK was in the process of retaining EU law following its departure from the EU [120]. This included talks regarding the possibility of EU law continuing to only apply in Northern Ireland but not in the rest of the UK [121]. Therefore, we excluded these laws to ensure consistency. While this decision resulted in the exclusion of some laws that would have otherwise been included, such as legislation regulating the transport of dogs within the UK and across borders, we were unaware of any a priori reasons why this would change the overall findings. Sixteen laws were excluded as originating from the EU, thirteen of which involved the transport of dogs.

Inclusion criteria were applied during the coding process detailed below. Sections were included if their contents would materially impact an average dog owner. This usually involved a section introducing a new offence or requirement. Any sections that related to dogs trained for specific purposes, such as police or assistance dogs (also referred to as service dogs in USA), were excluded. This was because the experiences of these dogs and their owners/handlers often differ from those who keep dogs primarily for companionship, and there is additional legislation that only impacts these working dogs [122,123]. Sections that were about the procedures, punishments, or enforcement of an offence were excluded as administrative. We excluded schedules from analysis because these are not supposed to introduce any new material changes, and instead provide additional details to the main sections of the legislation [124]. Law is a living document, and many amendments are made over the years. To ensure we avoided double coding, we excluded legislation that created such amendments so that we could code the sections within the context of the law being changed. (Please see File S1 for more information on the exclusion criteria and how it was applied during coding.)

**Figure 1:**
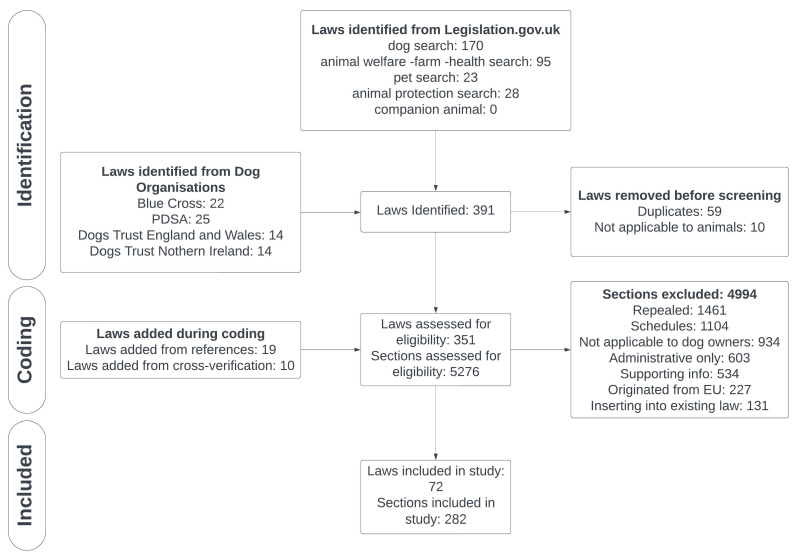
Flow diagram of the creation of the dog law database. Please refer to Supplementary File S2 for a step-by-step explanation of how these numbers were calculated.

After applying all exclusions, the final dataset consisted of 282 sections, representing 5% of all identified sections, from 72 laws, accounting for 20% of all identified laws. The systematic search identified 60% (168) of included sections. Dog organisation guides identified a further 54 (19%) sections, and the remainder were identified through the laws’ references (34 sections) and cross-verifying laws between the nations (26 sections). See Figure 1 for a flowchart detailing the search strategy, exclusions, and coding process. The full list of laws identified from the various sources is available in File S2.

### 2.2. Creation of a Framework to Compare Legal Jurisdiction

We developed a systematic framework, using content analysis, that for the first time enables the comparison of the benefits groups receive from law across areas of dog ownership. We defined benefits as increasing the mental, physical, and/or financial wellbeing of a group. We included five stakeholder groups most commonly impacted by dog-related legislation: the individual dog being targeted by the legislation, the rest of the dog population not being targeted, the dog’s owner, the general public, and the environment. For example, a law that requires dogs to be vaccinated for rabies impacts the individual dog that receives the vaccination, the dog’s owner who is required to pay for the vaccine, and other people, wildlife, and dogs, who are all less likely to contract rabies as a result. We split dogs into two groups, because some sections were aimed directly at individual dogs, while others were applicable to the dog population. At times, the interests of the individual dog and wider dog population did not align, and this approach enabled us to capture this. We also included the environment, which includes wildlife and eco-systems, because of the increased focus on the negative impacts dogs can have on the environment [92,125,126].

We also coded the area of dog ownership the law was targeting. These law areas were created by taking categories used by dog organisations’ public facing materials [114,115,116,117]. Examples include dog control, identification, animal welfare and nuisance (see File S1 for all categories with definitions). After initial analysis, it was decided to group law areas based on their main purpose, because many law areas overlapped and often represented an overarching goal. For example, dog welfare and cruelty often overlapped, because both categories aim to protect dogs, mostly from their owners. Therefore, we created four grouped law areas to best represent the data: Managing Dogs in Public, Dog Protection, Economic Activity. and Transporting Dogs Across Borders. Managing Dogs in Public incorporated the areas of identification, dangerous dogs, breed specific legislation, dog control, dog faeces, and nuisance. We adopted the concept of the public dog used in Carter [48] to decide upon the grouping. For example, nuisance was included here despite often occurring in the home because Carter [48] argues that once a dog impacts someone outside the family, they move from the domestic to the public domain and are treated as such. Dog Protection incorporated animal cruelty and animal welfare areas. Providing dog services and breeding and selling dogs were included in Economic Activity. Transporting Dogs Across Borders was not grouped because this area did not overlap with others.

We chose content analysis as the basis of this framework after conducting a literature review on the different methods used to extract meaning from interpretable text. Content analysis is a systematic methodology that analyses text to identify trends and word patterns. It is flexible enough to enable comparison of highly interpretable laws [127,128]. We developed our themes (law area and law benefits) deductively because we wanted to conduct a research question-led study [129]. However, we used pilot studies to develop these further and ensure they properly reflected the data [127].

### 2.3. Testing the Framework

To test the reliability and validity of the coding scheme, we conducted two pilot studies using a consensus approach, which involves discussing disagreements among coders until consensus is reached [130,131]. SAW recruited a master’s student (LMcD) who had previously worked as a property lawyer, and who was studying human psychology, to test the reliability of the coding scheme. After SAW created the dictionary that detailed how to code the data, SAW trained LMcD on the process and discussed the aims of the study. For each pilot, a random subset of laws was selected, and we coded these according to the dictionary. Once complete, we computed percentage agreement and Cohen’s Kappa to test the reliability of coding and discussed disagreements in depth. While there are disagreements over the use of reliability statistics in qualitative research [131,132], we used them to improve the creation and comprehension of the dictionary and guide in-depth discussions of disagreements. Reliability overall was low to adequate (see File S3 for coding reliability results), but generally improved across the pilot studies. Common disagreements included misunderstandings of the dictionary and differing opinions of the purpose of the law. After discussing the results, we updated the dictionary, and performed another round of piloting until any disagreements were due to our interpretation of the laws rather than misapplications of the dictionary. See File S1 for the final dictionary used for coding the main dataset.

### 2.4. Coding the Data

SAW and LMcD both coded the full dataset independently because there was low to adequate reliability in the pilot studies. This indicated that our backgrounds and experiences impacted our interpretation of the laws and that our impressions were not interchangeable [128]. We read each section and first decided if it met the inclusion criteria detailed in File S1. If included, we decided upon at least one law area such as dog control or dog welfare, then examined each stakeholder group to determine if the section applied to them, and if so, whether they benefited. We recorded a “yes” if the group benefited from the law, a “no” if they did not but would be impacted, and “no impact” if they would be unaffected by the law. We also recorded the reasons for our decisions and any thoughts we had about the laws and how we interpreted them.

The inter-rater and intra-rater reliability of coding the main datasets were assessed using percentage agreement and Gwet’s AC1. Due to the presence/absence nature of the data, with some categories having a high prevalence of absences, Gwet’s AC1 was deemed most appropriate to test reliability [133,134]. For inter-rater reliability, SAW and LMcD’s datasets were used in the analysis. To test intra-rater reliability, both raters recoded a random selection of sections (SAW recoded *n* = 334 and LMcD recoded *n* = 121).

Overall, results indicate that there was an adequate to good level of inter-rater reliability for the coding criteria. We had high reliability when applying the inclusion criteria with a percentage agreement of 96% and a Gwet’s AC1 coefficient of 0.95. The key disagreements were a result of some misapplications of the dictionary and differences in interpretation about what constituted administrative sections and sections that did not apply to dog owners. Our reliability when coding benefits ranged between 0.60 to 0.67. An exception was the benefits for the human population, which had a Gwet AC1 coefficient of 0.33, indicating low reliability. More than half of these disagreements were a result of differing opinions on how animal welfare legislation benefits the general public. LMcD believed that the general public’s mental health improves from knowing animals are being protected by the law. SAW disagreed with this, because the relevant sections and their accompanying explanatory notes did not explicitly reference the protection of human well-being, and no provisions were made to protect vulnerable groups, like family members or children, from those convicted of animal abuse charges (despite animal abuse being also a known predictor of abuse against humans [135]). This was interpreted as an indication that law-makers did not intend for the legislation to address the direct or indirect impact animal cruelty can have on human health and welfare.

For the intra-rater reliability assessment, both coders tended to have high levels of reliability for most categories. SAW was reliable in applying the inclusion criteria with a percentage agreement of 96% and a Gwet’s AC1 of 0.95. LMcD was less reliable with a percentage agreement of 85% and a Gwet’s AC1 of 0.77; however, this was still adequately reliable. The key cause of difference for both coders was deciding if a section was administrative. For the benefits categories, SAW had AC1 coefficients ranging from 0.45 to 1 with five of the seven categories above 0.9. Coding the benefits afforded to the dog population was the least reliable, with a coefficient of 0.46. LMcD’s AC1 coefficients ranged between 0.69 to 0.94. Interestingly, when one rater’s reliability was low, the other’s was high. Therefore, when creating the final dataset, in cases of disagreement, the decision of the rater with higher reliability was prioritised. See File S3 for all reliability results.

The final dataset was created by combining the two independently coded datasets. When both coders agreed on a code, this response was added to the final dataset. To decide on the final codes, SAW reviewed the reasons created during coding and the intra-rater reliability results. The decision for most cases was clear based on the coders’ recorded reasoning. A major exception to this was dangerous dog exemption legislation. These laws were designed to prevent banned dogs from being euthanised under the earlier dangerous dog legislation by creating restrictions that could severely impact on the dogs’ and owners’ quality of life. These restrictions would normally be coded as not being beneficial for individual dogs or owners, but within the context of the law’s creation, they allow for a dog to live, and so the amendment could be seen as a benefit. It was decided that these were benefits to dogs and their owners, because the scheme allowed for dogs to avoid automatically being euthanised [79]. Because laws were also identified while coding, there were some laws that only had one coder. These represented 18% (52 sections) of the final dataset. SAW reviewed these to make sure they were coded consistently and in line with the rest of the dataset (see File S2 for the final dataset).

During coding, it was apparent there was almost always equivalent legislation across the nations under study. Therefore, after creating the final dataset, we conducted cross-verification of sections across the nations. This was to identify any laws missing that should have been included, and also to validate the coding. First, we checked that the coding was consistent for any sections that were the same across the nations. For example, we matched Section 175 of the Road Traffic (Northern Ireland) Order 1981 and Section 170 of the Road Traffic Act 1988 that applies in Scotland, England, and Wales, because both laws create a duty to notify the police if a person injures an animal with their vehicle [136,137]. If we did not have a section for an offence/requirement that existed in other nations, we searched for an equivalent. This process identified an additional 26 sections to be included from 10 laws. To validate the law area and benefits coding, we compared the codes for each offence/requirement. We found that 82% of benefits coding and 93% of law area coding was coded consistently if the offence/requirement was the same across the nations.

### 2.5. Analysing the Data

Percentages were calculated to understand the prevalence of different grouped and ungrouped law areas. This was calculated as the number of sections that were coded as a law area, such as identification, dog control or transport, divided by the total number of included sections. Percentages were also calculated for the benefits afforded to each group. Frequencies of each code (benefit, disadvantage, and no impact) was divided by the total number of included sections. This was repeated for each of the stakeholder groups. To assess the differences in benefits for each group across the consolidated law areas, we divided the frequency of benefits of a stakeholder group by the total number of sections in each consolidated area. This process was conducted at a UK level and then computed for each nation to enable comparison. All calculations were conducted in Microsoft Excel.

## 3. Results

### 3.1. UK Level Results

#### 3.1.1. Areas of Dog Ownership

**Figure 2:**
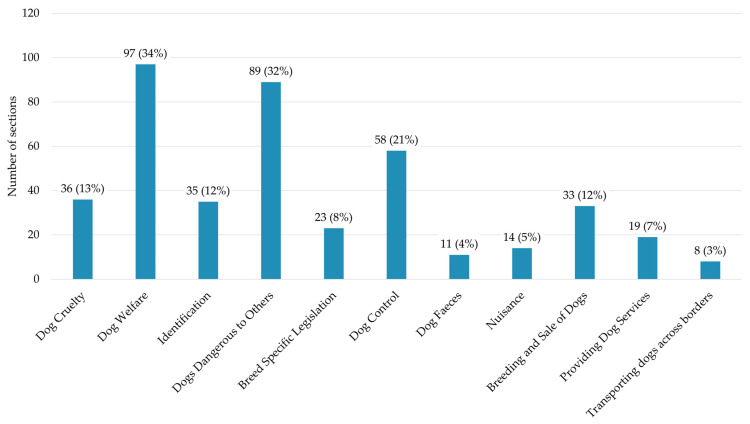
Number and percentage of included sections that related to areas of dog ownership. Dog Welfare and Dogs Dangerous to Others dominated legislative focus. Please note that some sections were applicable to multiple categories and therefore appear in more than one category.

The most common forms of legislation were those that managed dog welfare (97 sections, representing 35% of included sections) and dogs being dangerous to others, particularly to people and livestock (89 sections, representing 32% of included sections). Dog control, which included any sections that control or manage dogs when in public spaces, particularly managing stray dogs, was the only other area of dog ownership that received a relatively notable degree of attention with 58 sections, representing 21% of included sections. All other areas of dog ownership received relatively little focus in comparison; see Figure 2 for full results. Sections that dealt with transporting dogs across borders received the least attention, comprising just 3% (8) of sections. This is a result of most legislation managing this issue originating in the EU, and thus not being included in this study. Dog faeces was also very rarely legislated, with just 4% (11) of sections managing the issue. This likely results from the issue being managed locally, as 7 of the 11 sections granted powers to local authorities or councils to manage dog faeces.

When these areas of dog ownership were consolidated, the most legislative effort went towards Managing Dogs in Public, with 169 sections accounting for 60% of included sections. This group consisted of identification, dogs being dangerous to others, dog control, breed specific legislation, nuisance, and dog faeces. Dog Protections, created from Dog Welfare and Cruelty, was the next most common legislation, with 107 sections (38%). Few sections legislated Economic Activity (42 sections, representing 15% of included sections) or Transporting Dogs Across Borders (8 sections, representing 3% of included sections).

There were 44 sections (16%) that legislated for more than one of these areas. The majority of these cases (28) were a result of sections that regulate the breeding and sale of dogs, which are included in both the Economic Activity and Dog Protection groups. Economic Activity was rarely coded on its own, with only three sections not also being included in other areas. This overlap indicates that the focus of legislation managing the breeding and sale of dogs is mostly focused on ensuring the welfare of the dogs and puppies involved rather than protecting dog owners as consumers (the only exception being the Sale of Goods Act 1979 and the Consumer Rights Act 2015, which protect owners’ purchases). The other consolidated areas rarely overlapped. Only 13 sections of the 169 sections regarding Managing Dogs in Public were also coded in other areas (11 of these required dogs to be microchipped, in part to aid the reunification of dogs and owners).

#### 3.1.2. Law Benefits

**Figure 3:**
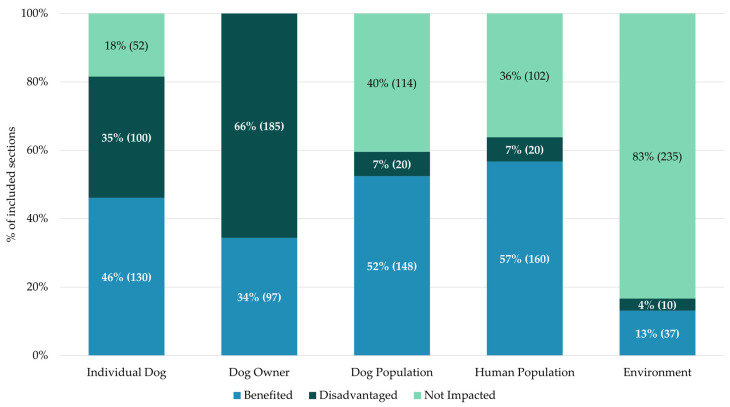
Comparison of the impact of sections on stakeholder groups.

The human and dog populations received the most benefits from legislation across all law areas and were rarely deprioritised, particularly the human population (see Figure 3). Only 20 sections (7% of included sections) disadvantaged the human population, with the remainder having no impact (102 sections, representing 36% of included sections). Almost half of the included sections (130 sections, representing 46% of included sections) benefited dogs who were being targeted by legislation (individual dogs). They rarely were not impacted by the sections, and instead were almost as likely to be disadvantaged as benefited by the law (100 sections, equating to 35% of included sections). Dog owners were disadvantaged the most out of all groups, and were only benefited in 34% of sections (97 sections). They were always impacted by the legislation, and so the remaining 185 sections (66%) disadvantaged or restricted their actions in some way. Finally, 83% (235) of sections did not impact the environment. The remaining sections tended to benefit the environment (37 sections representing 13% of included sections). All but two of these sections benefited wildlife. These benefits were derived from protection from those hunting with dogs (19 sections), preventing the spread of rabies (13 sections), or reducing the risk of unintentional poisoning (3 sections).

#### 3.1.3. Benefits by Consolidated Law Area

**Figure 4:**
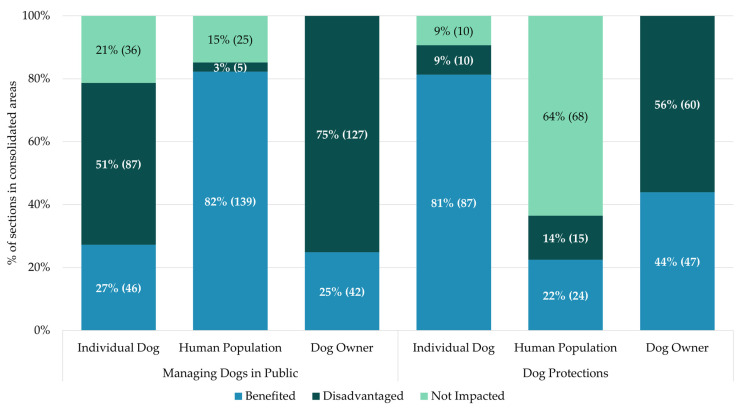
Percentage of benefits ascribed to stakeholder groups within consolidated areas of law. The impact of sections on stakeholder groups depends on the legislated area of dog ownership.

The benefits that groups derive from law depends on the area of dog ownership being legislated. This was especially the case when comparing legislation that dealt with dogs when they were in public versus private spheres of life. Sections that manage dogs in public almost always benefit the human population (139 sections, representing 82% of sections). They were only disadvantaged in five sections (three of which provide legal defences in civil court for dog owners, with the other two requiring people to inform police if they injure a dog in a car accident). In contrast, individual dogs were only benefitted in 27% (46) of sections when in public, and were disadvantaged in 51% of sections (87). Dog owners were predominately disadvantaged when in public with their dogs, being disadvantaged in 75% (127) of sections (see Figure 4).

The opposite pattern was found when dogs were being protected, often in the private sphere. Unsurprisingly, dogs benefited from 87 (81%) sections that aimed to protect them. Individual dogs were disadvantaged in 10 (9%) sections, which were often a result of protecting the wider dog population from an individual dog. For example, five of these sections were measures to prevent the spread of rabies. The dog being targeted either could be isolated or destroyed if found to be at risk of rabies for the benefit of other groups, including other dogs. In contrast, the human population was only benefitted in 22% (24) of sections. However, unlike when dogs are in public, the human population was mostly not impacted and only disadvantaged in 15 sections. Dog owners were benefited and disadvantaged in relatively equal measures. They benefited in 44% (47) of sections and were disadvantaged in 56% (60) of sections. The other groups were impacted to a similar degree across the consolidated areas (see File S4 for full results of all groups).

### 3.2. Differences Across Nations

#### 3.2.1. Patterns of Law Making Across the Nations

There were similar numbers of sections across the nations. Of the 275 sections included in the study, 101 sections (37%) included were in force in England, 101 sections (37%) in Northern Ireland, 95 (35%) in Scotland, and 106 (39%) in Wales. Legislation in England, Scotland, and Wales was mostly created by primary legislation. In contrast, Northern Ireland was more likely to use secondary legislation, possibly influenced by periods when the Northern Ireland Assembly (and its various predecessors) was not in session [138]. See Table 1 for an overview.

**Figure 5:**
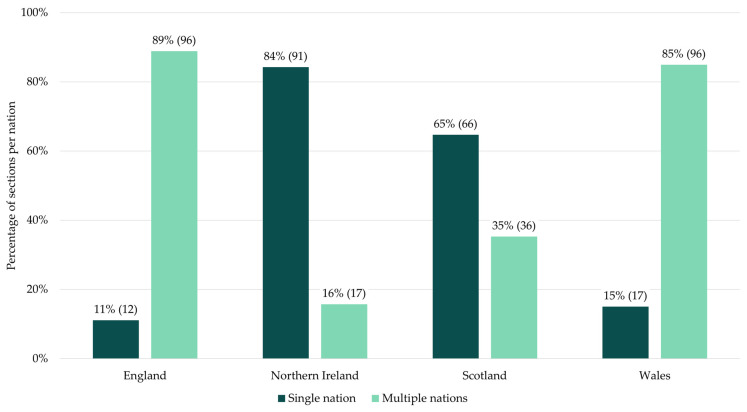
The number and percentage of sections that were created to apply in only one nation versus those created for more than one nation. England, Scotland, and Wales shared most laws, while the majority of laws in Northern Ireland were only in Northern Ireland.

The extent to which the devolved nations created legislation solely for their own nations varied (see Figure 5). Almost all of Northern Ireland’s sections only applied in Northern Ireland, while the central UK Government created most of the sections that applied in England and Wales. While the central UK Government is the only legislative body able to create English laws, agriculture is a devolved issue in Wales, but the Welsh parliament made no primary legislation relating to dogs. Approximately two-thirds of sections in force in Scotland only apply in Scotland. The remaining 35% of sections (36) were created before devolution was established in Scotland in 1998 [139]. There were only three laws that were created for all nations in the UK. These were the Veterinary Surgeons Act 1966, that creates a professional registry of veterinary professionals, and the Sale of Goods Act 1979 and Consumer Rights Act 2015, which protect dog owners as consumers when buying dogs.

#### 3.2.2. Differences in Law Area and Benefits Across Nations

**Figure 6:**
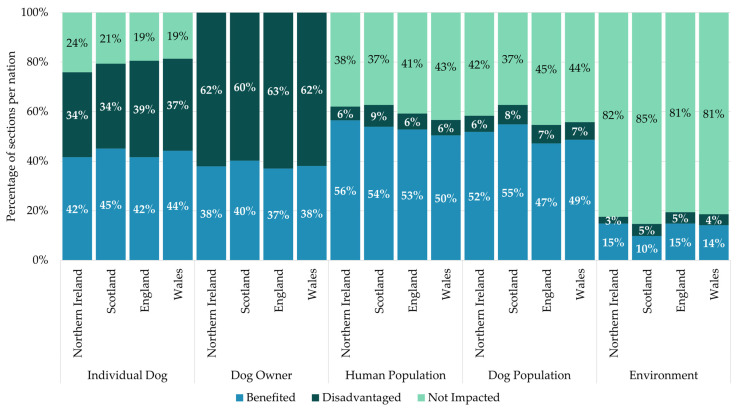
Comparison of the percentage of sections in each country that benefited, disadvantaged, and did not impact stakeholder groups across nations.

Despite differences in how legislation was created across the nations, there were few differences in the areas of dog ownership being legislated (see Table 2) or the number of benefits received by stakeholders (see Figure 6). Of the eleven law areas, seven had less than a 5% difference in the number of sections between the nation with the most and the nation with the fewest coded law areas. The largest difference was sections managing dogs being dangerous to others. Northern Ireland had 15% (14) fewer sections compared to England, who had the most (39 sections, accounting for 38% of English sections). This difference was a result of Northern Ireland not passing any legislation that bans hunting with dogs, unlike the rest of the UK. The only other major difference was sections that dealt with dogs being transported across borders. All eight sections were only in force in Northern Ireland. The key legislation that manages this issue originated from the EU and so was excluded from our analysis. Northern Ireland was the only nation to have older legislation that was retained alongside the EU legislation. As a result of these similarities in the issues being legislated, the sections impacted the stakeholder groups in similar ways across nations. This resulted in few differences in how stakeholder groups benefited (see File S4 for full results of the comparison across nations).

## 4. Discussion

Our findings suggest that conflicting ideas about dogs continue to be reflected in the UK legislation. Protecting dog welfare and controlling dogs, especially potentially dangerous ones, was highly legislated. Although dogs are increasingly described within culture as humans, children, and family, the law does not reflect this. Instead, the legislation aligns with the complicated, often contradictory way Western societies think of dogs and animals more generally [19,140]. Dogs are legislated as property, as well as being entitled to protection from their owners, while also being seen as potential threats requiring others to be protected from them. Owners are at the centre of this arrangement, resulting in dogs being unable to exist outside of the ownership of humans [24]. This tension between loving and fearing dogs suggests we continue to live in the shadows of Victorian ideas of dogs.

However, there are some signs of change. In 2020, Scotland amended the Animal Health and Welfare (Scotland) Act 2006 allowing animals to be taken from their owners in cases of suspected welfare or cruelty offences and rehomed. In the rest of the UK, a dog can only be seized and rehomed with the approval of the courts, which is often a lengthy and expensive process. Scotland’s amendment erodes the property rights of owners while solely prioritising dog welfare [66]. More recently, and after our data was collected, England and Northern Ireland passed the Pet Abduction Act 2024, which created specific penalties if a cat or dog is stolen. Previously, the theft of companion animals was treated as the theft of inanimate property, dependent on the pet’s monetary value [141]. Northern Ireland’s agricultural minister Andrew Muir announced that the law was to ‘recognise that [dogs and cats] are not mere property but sentient beings’ [142]. This act elevates the status of dogs and instead recognises the emotional impacts on the dogs and owners involved. While these examples suggest that the law is increasingly aligning with how many in the UK view companion animals, they also emphasise dogs’ need for governments to enact distinct laws to recognise their status as having sentience and special interests.

Another interpretation of the equal attention placed on dog welfare and dangerous dogs is that the law is successfully balancing the needs of dogs with the safety of the public. However, we found that dogs were only prioritised in the private sphere, but deprioritised in public, supporting what Carter [48] argued as dogs now leading separate public and private lives. ‘Responsible dog ownership’ campaigns aim to encourage owners to meet their dog welfare and control requirements, often blurring these concepts [53,143]. However, these results suggest that advertising control measures as improving dog welfare might be misleading. It might also be why research has found that dog owners tend to describe themselves as responsible even if they are not meeting dog control requirements [53,54]. Westgarth et al. [53] found that dog owners primarily used the term to describe the responsibility they felt towards their dog. Therefore, responsible dog ownership may not be an effective vehicle to encourage dog owners to comply with dog control measures. Further research is required to evaluate the effectiveness of ‘responsible dog ownership’ campaigns in encouraging dog owners to meet their legal obligations.

Creating separate public and private lives for dogs also means that the law is not reflecting dog owners’ familial feelings towards their dogs. This might be driving conflict in public spaces. Studies found that the underlying cause of local conflicts was often the differing belief in who should be prioritised in public spaces [22,50,51]. We found that the UK government aligns with those opposed to dogs, overwhelmingly benefiting the general public. This might not be reflective of the UK public’s opinions of dogs. For example, YouGov [144] found that 62% of Britons believed it was acceptable for dogs to be off lead in parks. However, there was a divide in the perception of the behaviour of dog owners, with non-owners less likely to believe that dog owners were considerate. This aligns with research that dog owners tend to prioritise their dog’s safety and wellbeing over other groups [53,55,145]. As people tend to believe that the law aligns with what they think it should be [146], and research suggests knowledge of dog law is low [99], different populations could be behaving in ways they incorrectly think is following the law, and thus potentially creating conflict [22]. Future research could investigate the knowledge and opinions of private and public laws using a nationally representative sample to better understand whether current law aligns with the majority of people’s views.

This public/private divide could also make it challenging for dog owners to meet their dog’s welfare needs. Dog owners only benefited in a third of all laws, mainly because the majority of sections increased owners’ legal responsibilities both in the public and private spheres. However, when viewed holistically, the law does not always align across areas of dog ownership, potentially making it hard for owners to meet all of their legal responsibilities. Dog owners are advised by the UK devolved governments that to meet the requirements outlined in their respective animal welfare acts, dog owners must provide their dogs with sufficient exercise, company, and socialisation with people and dogs [147,148,149,150]. Meeting these needs requires access to public space for most people, especially for those who work outside their home. If dogs and their owners receive few benefits when in public spaces and are deprioritised, owners are unlikely to effectively meet their dog’s welfare needs.

This is especially important because loneliness, obesity, and inappropriate socialisation have been identified as key issues facing companion dogs [151,152,153,154]. On the face of it, increasing the incorporation of dogs into public spaces could help with these issues. Evidence suggests that dog owners would like to bring their dogs with them during their daily routines, including bringing them to work, shops, and cafes [22,38,155]. This may also help dogs emotionally, because dogs who experience a wider variety of experiences are less likely to experience anxiety [156,157]. Meyer et al. [158] also found that daily off-lead walking was associated with a lower risk of aggression and disobedience. A large proportion of dogs exhibit some form of behaviour problem, such as fear, disobedience, especially jumping up, anxiety, or aggression, with reports of these behaviours growing each year [158,159,160,161]. As dogs’ acceptance into public spaces depends on them behaving, as described by Instone and Sweeney [50], as adult humans, it seems unlikely that opening public spaces to dogs en masse will be successful.

In place of incorporating their dogs into their public lives, dog owners are increasingly using dog services such as dog walkers, ’doggy daycares’, or dog ‘borrowing’ services to look after their dogs while they are away [162,163]. We found that there was relatively little legislation regulating these areas, with only 19 sections for all four nations. England was the only nation that specifically regulated dog home boarding services and daycares. These regulations have created specific requirements, such as when crates can be used, how long a dog can be kept in one, and how different dogs can interact. There is less certainty over how the other nations manage these services. Their current legislation, created in the 1960s and 1970s, discuss requirements for kennels with a specific focus on the prevention of the spread of disease. The Scottish Government [164] have indicated that they plan to update legislation to bring it in line with England’s approach. There is more uncertainty in Northern Ireland, with the agriculture minister in 2022 reporting that home boarding was not covered by legislation, but as money is exchanged for services, these businesses must have a licence [165]. It would be beneficial for these laws to be updated to reflect the realities of how these services are increasingly being delivered to further protect dog welfare.

However, as dog ownership continues to grow and more dog owners wish to bring their dogs into public, proactive solutions from government are required to reduce problem behaviours. Local solutions that aim to work proactively to prevent dog bites, such as the ‘LEAD’ program in England or the ‘Calgary Model’ in Canada, have demonstrated some success [57,166]. These programs focus on control and quickly intervening when any incident occurs, including those that many police forces would previously not be involved in. This includes fining and/or educating the owner, placing control conditions on the dog, and promoting ‘responsible dog ownership’ practices. However, these approaches do not prevent undesirable dog behaviours from occurring in the first place. National governments are best placed to introduce national legislation that can address the root causes of the development of undesirable behaviours that are not dependent on dog owners’ ability to control their dogs.

The breeding and sale of dogs has received widespread attention from media, charities, and governments because of the welfare impacts they have on dogs [167,168,169,170]. However, how dogs are bred and sold, from the selection of parents to the eventual sale, can also impact the behaviour of dogs. Factors such as being bred in high volume breeding facilities (commonly known as puppy farms) [171], being sold at earlier ages [172], and having aggressive or anxious parents [173,174,175] are associated with increased problem behaviours. There is also a high prevalence of dogs bred with inherited disorders or aesthetic features that can cause severe, painful health issues [176,177]). As pain is increasingly recognised as a potential driver of undesirable behaviours [178,179], this might not only be a welfare issue, but a public safety one too. We found that there were relatively few sections that regulated breeding and selling dogs, despite it being named as a top priority by governments [180,181,182]. These sections tended to focus more on the early life experiences of puppies and, in England and Scotland, the welfare of the mother. The UK’s breeding laws have come under scrutiny, with calls for greater protections to promote the breeding of healthy and well-adjusted dogs [170,183,184]. Framing the issue as a public safety intervention, rather than solely an animal welfare one, might prove more persuasive with a government that has historically prioritised human safety.

Despite some small differences in the content of the sections, we found no differences in who benefited or the type of laws being created across the four nations. This was unexpected because the purpose of devolution is to transfer power to local governments to make decisions (and laws) that are more aligned with the people and communities they govern [139]. This result is likely due to the nations replicating policies introduced elsewhere if they prove to be effective, leading to largely similar legislation across the UK [139]. When validating the data across the nations, we found that most provisions were found in some form across the UK, sometimes being word for word the same. Future research could assess whether there are differences in public opinion of the laws across the nations to better understand if this approach represents national sentiments.

### Future Directions and Limitations

This study was the first of its kind, and so there are many potential avenues future research could take. Future research could replicate this study in other countries with different government structures and legislative approaches. This would allow results to be compared to create a better understanding of how dogs are being legislated across countries. It can particularly help to identify gaps in legislation. For example, outdoor chaining of dogs is not legislated in any of the UK nations, unlike most other culturally Western countries [77,185]. None of the UK nations’ code of conducts that advise owners on their responsibilities discuss chaining or tethering of dogs [147,148,149,186]. It is unclear if this is not legislated because the practice is uncommon, or if this requires further scrutiny in the UK. The questions raised from the absence of legislation in one nation in contrast to others can direct future research and legislative strategies.

Cross-country comparisons can also provide a deeper understanding of cultural differences in the perception of dogs and how these perceptions influence dogs’ management through legislation. Dogs hold different meanings across countries, and they can be complex and changing [40,187,188,189,190,191,192,193,194,195]. Studies indicate a rise in perceiving dogs as family and children in countries who have different dog histories and cultures, such as India [40], Japan [194], and China [193]. Comparisons with countries who have different dog histories and cultures which may have led to different legislative approaches would be especially interesting. For example, India categorises dogs into owned dogs and street dogs, legitimising dogs’ existence in public spaces, which differs from the UK’s approach [24,196]. Future studies could compare countries to identify if laws are shifting alongside changes in perceptions of dogs. As each country has different legislative systems. with law-making conducted at different levels of government, the framework can enable cross-country comparisons. Adaptation of the framework could facilitate fruitful comparisons between nations whose law-making incorporates supranational processes (such as within the EU), as well as among distinct legislative regions within nations (such as US states).

Future research could also split up the stakeholder groups to gain more insight into who tends to hold power in society. For example, laws that managed dogs around livestock were common in dog control legislation. These laws aimed to specifically protect farmers rather than the public in general. Similarly, legislation controlling hunting with dogs often was aimed at targeting specific activities that only certain groups would partake in (for example, badger baiting or hunting with hounds). Surprisingly, Northern Ireland legislation often provided specific benefits to those who partake in hunting, suggesting these groups hold some power in society. Therefore, future studies could split up the general public group into special interest groups to better understand the potential degree of power they hold.

Additionally, dogs occupy a wide range of roles in human societies, and so future research could focus on specific groups of dogs used for different purposes. In this study, we focused on dogs who were primarily kept for companionship, and so excluded working dogs such as assistance and police dogs from analysis. This was because these dogs require specialised training and have specific legislation applying only to them. Future studies could focus solely on working dogs, especially assistance dogs, where a lack of consistent legislation within and across countries has negative impacts on a range of stakeholders [122]. The framework could allow for systematic comparisons of law that can highlight issues that legislation might be contributing to. For example, the United States has had highly publicised issues regarding emotional support dogs and fraudulent assistance dogs gaining access to public spaces, including shops, airlines, and rental accommodation [197]. This has led to increased legislative focus, including passing amendments to more narrowly define what constitutes an assistance, service, and/or emotional support animal [197,198]. However, in the UK, all nations define assistance dogs as those trained to perform specific tasks. As a result, emotional support dogs, which do not require specialised training, are not recognised as assistance dogs, and are not entitled to accommodations from businesses and organisations [199]. Future studies can use our framework to examine how legislation could be clarified to reduce conflicts in society.

This framework could also be applied to other species, especially those whose primary role has changed, like cats [200,201] and horses [202], or is currently changing, like ‘backyard’ chickens [203]. Their changing role may also impact how they are legislated as these species are kept in new ways or begin to affect other people and animals. Cats, like dogs, are seen by many as family members, but the impacts they have on others, particularly wildlife, has raised greater concerns over how they should be kept [42,200,204]. They are also receiving more focus in legislation, with microchipping becoming mandatory in England in 2023, and Northern Ireland expected to introduce similar legislation in 2025 [180]. Horses have also moved status from primarily working and leisure animals to pets, with many horse owners forming strong emotional bonds with their horses [205]. They can also be classed differently in legislation based on their function and so, like dogs, are impacted by a wide range of legislation [206]. Future studies could use the framework to identify gaps in legislation and ensure future laws work holistically.

A key limitation of the study is that all benefits coded were not equal. To be conversative and increase coding reliability, we coded a section as benefiting a group if there was at least one aspect that positively impacted them. This may have overemphasised the true benefits some groups received. For example, we coded the dangerous dog exemption schemes introduced in 1997 that granted courts the ability to prevent banned breeds from being euthanised as protecting dogs and dog owners. This is despite the strict conditions required that would likely reduce the quality of life of these groups. Although the alternative within the context of the law is the dog’s death, these benefits may not be felt by these groups in their daily lives, and the restrictions may still cause harm to the dogs affected [79]. Future research could be more restrictive when defining what it means for a group to benefit from a law.

This study only analysed the law created by government bodies, and did not take case law or the law’s implementation into account. This was to understand the rules that dogs and owners are expected to follow rather than how they are interpreted by the courts. While we found few differences in laws across nations, these laws may be interpreted differently both by the courts and government departments tasked with implementing them. Future studies could assess if there are differences in how different areas of law are being implemented by courts and government departments across the nations.

This framework was created using a country that largely follows a common law system (Scotland is a mixture of common and civil law [207]) but uses legislation as the primary method to introduce new laws [111,208]. The creation and sources of legislation can vary across countries, shaped by differences in legal systems, government structures, and the extent of religious influence [209]. It has been argued that the differences between the two most widespread systems, common law (judge-made law) and civil law (codified law), have been diminishing as both increasingly rely on legislation as the primary method of lawmaking [207,208]. This means that this framework can likely be used for other countries with both civil and common law systems. However, the framework may be more challenging to apply to other forms of legal systems and government structures, such as religious-based systems, e.g., Islamic law, which have different and sometimes numerous sources of law [209]. Legislation may therefore play a more limited role in lawmaking, which may limit the ability of the framework to create a comprehensive understanding of how dogs are regulated. We encourage legal scholars working at the interface of human–animal interactions and other systems of law to adapt the framework to work with differing legal systems.

## 5. Conclusions

This study, for the first time, enabled the systematic comparison of dog laws across different governments, offering a holistic understanding of dog legislation within a country. Our results found differences in the areas of dog ownership that governments prioritise, as well as the benefits provided to stakeholders. The general public received the greatest benefits and were rarely disadvantaged in legislation. This suggests that campaigns advocating for stronger dog welfare legislation would benefit from framing their arguments around public safety. This approach is especially relevant in discussions concerning the breeding, sale, and importation of puppies and dogs. While dog owners are central to all legislation, expected to care for their pets’ welfare and ensure public safety, they receive few benefits and may require additional support to meet their obligations. Governments should aim to avoid blurring the concepts of dog control and welfare when promoting responsible dog ownership. As legislation that manages dogs in public disadvantage both dogs and owners, combining these concepts potentially leads to low compliance and increased conflicts. Future studies investigating the effectiveness of responsible dog ownership campaigns would be beneficial.

As a case study, these findings are likely influenced by the United Kingdom’s complex history and relationship with dogs. However, due to the impacts of colonialism, the results may be applicable to other countries, particularly Western countries with similar legal frameworks. Future research can build on this framework to explore countries with more complex government systems, facilitating cross-country comparisons that deepen our understanding of how dogs are managed globally.

## Figures and Tables

**Table 1:** The number of laws included in the study that were created either as primary or secondary legislation split by the country the laws extended to. All percentages represent the proportion of included laws in each nation.

Nation	Primary Legislation	Secondary Legislation	Total Legislation
England	20 (67%)	10 (33%)	30
Northern Ireland	11 (41%)	16 (59%)	27
Scotland	19 (70%)	8 (30%)	27
Wales	20 (63%)	12 (38%)	32

**Table 2:** Comparison of the percentage of included sections by areas of dog ownership across nations. There was little difference between law areas across the nations.

Law Area	Northern Ireland	Scotland	England	Wales
Identification	12% (13)	7% (8)	10% (10)	9% (10)
Dangerous dogs	23% (25)	31% (33)	38% (39)	35% (39)
Breed Specific Legislation	6% (7)	6% (6)	12% (12)	11% (12)
Animal Cruelty	8% (9)	14% (15)	11% (11)	11% (12)
Animal Welfare	32% (35)	30% (32)	29% (30)	31% (35)
Dog Control	19% (21)	20% (22)	23% (23)	20% (23)
Dog Faeces	3% (3)	5% (5)	4% (4)	4% (4)
Nuisance	4% (4)	6% (6)	7% (7)	6% (7)
Breeding and Selling Dogs	14% (15)	11% (12)	12% (12)	14% (16)
Providing Dog Services	8% (9)	10% (11)	11% (11)	10% (11)
Transporting dogs across borders	7% (8)	0% (0)	0% (0)	0% (0)

## Data Availability

Data are available as listed under Supplementary Materials (Files S1–S4).

## Supplementary Materials

The following supporting information can be downloaded at: https://www.mdpi.com/article/10.3390/ani15050647/s1, File S1: Final Coding Dictionary; File S2: Final Dataset and Systematic Search Results; File S3: Coding Reliability Results and Statistics; File S4: Additional Results.
