# Supplementary material for: The Creation of a Systematic Framework to Assess Dog Laws and Their Relationship to Societal Changes in the United Kingdom
Source: Animals (Basel). 2025 Feb 23;15(5):647. doi: 10.3390/ani15050647 (PMC11898190; doi:10.3390/ani15050647)
Supplement: Supplementary file 1 [file animals-15-00647-s001.zip › File S1. Coding Dictionary.pdf]

File S1. Final Coding Dictionary

This study aims to develop an understanding of the current legislative landscape for dogs and how they differ across nations. To test the differences in policymaking approach, we are coding primary and secondary legislation sections on two aspects, who the law is benefiting and the type of issue the law is addressing.

Scope and Inclusion Criteria

We are solely interested in the impacts of UK laws on the average dog owner who keeps their dog for non-commercial and non-specialist purposes. Therefore, only sections that change a dog owner's experiences are in scope. A section will be included if they impact:

- how a dog is looked after and owned
- how a dog is bought, sold or changes ownership
- the products or services an owner can use or buy in relation to looking after their dog
- how a dog and their owner interact with those around them, including the environment, animals and people

Only laws that are introducing a new offence or amending a previous offence that would constitute a material change for dog owners are to be included. Any sections that make a change that will only change how a law will be enforced or provide more clarity on the law are to be excluded. Examples include increasing a fine, detailing how offences can be enforced and creating an offence when the details have been outlined elsewhere. If any part of the section does make a material change to dogs or their owners, then it should be included.

Because we are not measuring the seriousness of offences but only if they exist, any section that introduces or changes a fine for an offence already introduced in previous legislation or elsewhere in the current legislation should be excluded. Similarly, a section that introduces the ability for a person to make an appeal will be excluded because it is not introducing an offence. Both of these examples would be excluded as administrative only.

Only laws that originated in the UK are to be included and so those that were first created in the EU should be excluded. Legislation that is made in the UK, but its sole purpose is to change UK legislation to reflect EU regulations should be excluded. These will usually have a European Commission Number (denoted as (EC) No 1523/2007 for example) somewhere in one of the sections or explanatory notes. If this number is found in one section, all sections in that law should be excluded.

Any schedules that are attached to legislation are to be excluded. Schedules are used to provide additional detail to the sections in legislation. Although Schedules have the same statutory effect as clauses, they are designed to provide additional detail that may detract from the sequential effect of the main body of the legislation (May, 2019). Therefore, because they are unlikely to make material changes to dog law, they are being excluded from the study.

Supporting information are defined as sections that are solely written to aid in the interpretation or understanding of the law as a whole. These are to be excluded because they are not making any material changes to dogs and owners. However, these should be read first to understand the language and context of the acts. Sections marked as EN (Explanatory Notes) are especially helpful as they provide an explanation of the aim of the law in non-legal language.

Sections are coded as supporting info if they:

- are marked as EN (explanatory notes)
- have citation, commencement and/or extent as the first lines
- have revocation included as the first lines of a section
- include the interpretation and/or definitions of terms to be used in the act
- include other information that is solely written to aid the understanding and interpretation of other sections in the act

If a section introduces a new offence but also includes supporting info, the section should be included. Only exclude when the entire section is defined as supporting info.

Exclusion codes

Below are the various codes that should be used when deciding if a section should be excluded. Only exclude the section if no material changes are being made.

Table S1. Codes used to explain why a section has been excluded.

Exclusion	Meaning	Why this is being excluded	Examples
Administrative only	Any section that provides no material changes for dog owners and their dogs. Administrative only sections include those that provide clarity on existing offences or legislation or provide more detail on how an offence should be enforced. The difference between supporting info is that changes are being made but they are immaterial for our purposes.	The material change has already been coded when the original changes were set out. Therefore, these details are more for authorities, courts and enforcement officers than dog owners. By excluding these we are also limiting the chance of double coding the same content.	Increasing a fine, detailing when blood tests should be completed if blood tests are required by previous legislation, allowing for an appeal, providing acceptable legal defences
Section repealed	The section has been repealed since its creation. Do not exclude if the section has been repealed in one jurisdiction but not the others.	The section has no bearing on the current lives of dogs and their owners and so therefore is no longer relevant	Textual amendment would say section repealed or revoked
Inserting into an existing law	Some sections in the legislation have been inserted through an amendment act made at	This is to avoid double coding	Textual amendment that says text inserted by law X and the brackets are

	another point in time. When a section says to insert XYZ into Act X then exclude using this code. Sections inserted should be coded in the context of the main act the section has been inserted into.		around the entire section
Supporting info	Information that relates to the understanding of the sections in an act or parent act that are creating a material change. These sections are expanding on how the rules will practically work. They are not making any changes.	These sections are not making changes to the lives of dogs and their owners. These sections are being read before coding to aid in the interpretation of the sections included in the study.	Commencement information, explanatory notes, extent
Not applicable to dog owners	Sections that relate to other stakeholders that do not involve enforcement officers, the courts or direct authorities. These stakeholders may include restaurant owners, air or sea authorities, large scale dog breeders, guard dog kennels or veterinary surgeons. Also excluded are sections that are designed to impact dogs trained for a specific function such as assistance, police and service dogs.	We wish to understand the expected impacts of these laws on the average pet dog owner and not the running of businesses or organisations that relate to dogs. Sections relating to these groups will only be included if the law changes the experiences of dogs and their owners when interacting with these stakeholders.	Sections that relate to the running of a pet shop, guard dog kennels, licensing requirements for a vet surgeon that does impact the services they provide to dog owners.
Schedule	Any section marked as a schedule.	Schedules are designed to provide more in-depth information on a section. Therefore, the information included here will not create any material changes to dog owners.	Sections that are listed as Sch x
Originated from the EU	Any section that originated in the EU. Includes any section that is only ratifying EU law in the UK.	We are interested in laws that were derived as a result of the UK's societal forces with the	Laws might include a European Commission number

		aim to better understand differences across nations.	e.g. (EC) No 1523/2007

Instructions

Enter a yes or no in the Inclusion field based on your assessment of the sections. Enter unsure and move on if you can't decide if it should be in scope or not. Add an exclusion reason using the codes above. If there is more than one reason for an exclusion, include the one that most likely excludes the section. For example, if a section is a schedule and also does not apply to dog owners, use 'schedule' because this will be excluded regardless of the content.

If you have decided that a section is out of scope, do not continue with coding. Exclude it with a reason and move onto the next section.

Coding Categories

Law Areas

This field is designed to categorise the sections into different areas of dog ownership. If a section is to be included, add at least one law area from the list below. If you are unsure of any write unsure before the area that you best believe fits the section, eg. Unsure Identification. If you cannot decide what code to use, write unsure.

Table S2. Definition of the law area codes.

**Law Area**	**Definition**
Identification	Any section that involves assigning owner information to a dog. Includes licenses, microchips, and DNA databases.
Dogs Dangerous to Others	Sections that involve dog attacks to people, livestock, wildlife or other animals, whether intentional or not.
Breed Specific Legislation	Sections that ban or restrict ownership of specified breeds. Includes any section in relation to the management, sale, care, or destruction of specified breeds.
Animal Cruelty	Sections that prevent unnecessary suffering of a dog. Does not include neglect and the lack of day-to-day care of a dog (these are under animal welfare). Usually involves someone committing an act that will cause suffering.
Animal Welfare	Sections that promote positive emotional states and avoid negative welfare. Includes banning of previously acceptable practices such as tail docking and the use of electric shock collars.
Dog Control	Sections that relate to the control or management of dogs in public spaces. These include sections that restrict or ban dogs from public spaces, mandate the use of a lead if directed to do so, limit the number of dogs in a public

	space at any time or penalise dogs being out of the charge or control of a designated person.
Dog Faeces	Sections that include any reference to dog faeces and its removal.
Nuisance	Sections that involve banning a specific natural behaviour of a dog from occurring, either in public or private spaces. Excessive barking is included. Being out of control is not included.
Breeding and Sale of Dogs	Any section that involves the passing of ownership of a dog from one person to the other or the breeding of dogs for sale.
Providing Dog Services	Sections involving the sale of a dog related service. This includes dog boarding establishments, guard dog establishments, pet cemeteries, veterinary services, dog walkers and doggy day cares.
Transporting dogs across borders	Sections that relate to dogs being transported across borders. Rabies legislation governing how dogs are transported in and out of the UK are included here.

Law Benefits

We aim to understand the differing priorities of law-making across nations by deciding who the sections are designed to benefit or disadvantage.

Benefits are granted when the intended effect of a section is to increase the mental, physical and/or financial wellbeing of a group. Both direct and indirect impacts will be taken into account.

Coding Instructions

When deciding how a section will impact a group, we are making decisions based on how we assume the lawmakers believed the section will impact groups. Only the explanatory notes of a law when available can be used to make a decision.

To determine whether a group will be benefited, use the flowchart below. Please add a short one-line reason why you made your decision.

Figure S1. Coding flowchart.

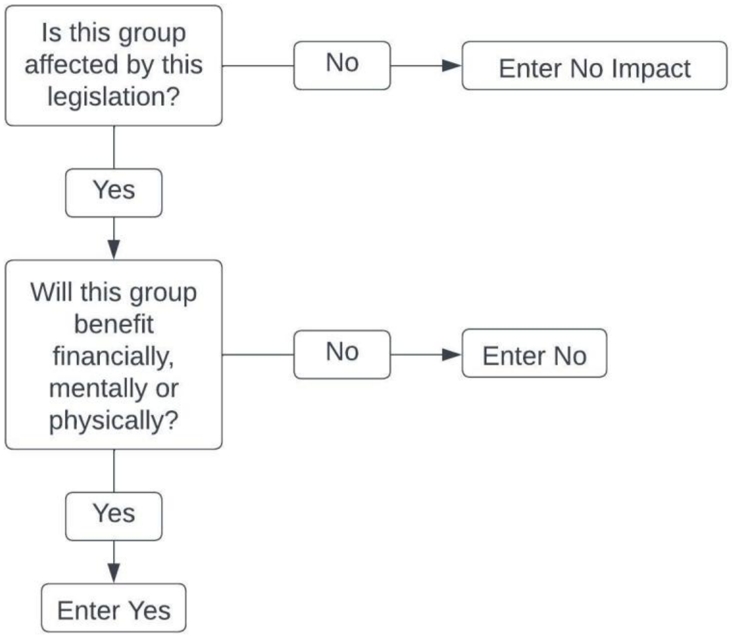

If there are multiple different issues being raised in a section, if a group is being benefited by one paragraph but not another, add a yes. We are interested in whether a particular group's wellbeing is being thought of, not the extent to which they are being protected.

For example, a section with one paragraph that says a dog should be destroyed if dangerous and another says it cannot be transferred to be scientifically tested on would be a yes for protection for the individual dog. Although they are likely to be destroyed which is a no to being protected, the lawmakers wanted to prevent suffering by preventing animal testing and so that would be a yes. One yes takes precedence over any nos.

We are considering 5 different stakeholder groups who may be impacted by legislation which are detailed in the table below.

Table S3. Definition of the stakeholder groups who may be impacted by legislation.

Group name	Data name	Definition
Individual Dog	ind_dog	The dog (or specific group of dogs) that are being targeted by the section or is being discussed in the section. The dogs' point of view is the central guiding force when deciding if they are being benefited, disadvantaged or not impacted.
Dog Population	dog_pop	The overall dog population living under the geographical extent of the legislation that are not being directly targeted. The dogs' point of view is the central guiding force when deciding if they are being benefited, disadvantaged or not impacted.
Dog Owner	dog_owner	The owner(s) of the individual dog being targeted by legislation. Can take the positive effects of dog ownership (increased comfort, companionship, increased daily exercise) and negative aspects of ownership (increased stress if dog is sick, has problem behaviour,

		financial costs of dog ownership) into account. If a section places increased legal responsibilities on an owner, add a no here.
General population	human_pop	People who live under the geographical extent of the legislation and will be impacted by legislation targeting other dogs that are not their own. **Includes potential impacts of legislation on owned animal such as livestock or companion animals** because these are deemed to be the person's property under the law and can impact a person's mental or financial wellbeing.
Environment	enviro	Any species of organisms in the Animal, Plant or Fungi Kingdoms that are unowned or considered 'wild'. Also includes the protection of ecosystems, biodiversity and public green spaces.

Coding Process

To start open the excel document and open the legislation on the legislation.gov.uk website.

1. Check if the law is repealed as a whole. If yes, enter exclusion yes and exclusion reason as **repealed.** If not, continue to 2.
2. Check details are correct per section and update if not correct (all geographic and version information was taken from the legislation level information and may not be correct for the section)
3. Complete coding for the section
4. If any of the coding is difficult and highly interpretable enter yes in the 'can be argued either way' column and add a short description of why it is and the various ways the code could be argued.
5. Add any other thoughts or if something is interesting into the 'Comment' box but add a no to the 'can be argued either way' column if it is not highly interpretable
