# Supplementary material for: The Creation of a Systematic Framework to Assess Dog Laws and Their Relationship to Societal Changes in the United Kingdom
Source: Animals (Basel). 2025 Feb 23;15(5):647. doi: 10.3390/ani15050647 (PMC11898190; doi:10.3390/ani15050647)
Supplement: Supplementary file 1 [file animals-15-00647-s001.zip › File S3. Reliability Results and Statistics.pdf]

File S3. Coding Reliability Results and Statistics

Main Study's Results

Table S1. Gwet's AC1 results for inter-rater reliability

Variable	n_Units	pa	pe	coeff.val	coeff.se	conf.int
Inclusions	4358	0.89	0.09	0.88	0.01	(0.87,0.895)
Individual dog	202	0.72	0.28	0.61	0.06	(0.495,0.725)
Dog population	201	0.69	0.17	0.62	0.05	(0.522,0.724)
Dog owner	202	0.66	0.14	0.60	0.05	(0.493,0.704)
Human population	201	0.45	0.18	0.33	0.06	(0.21,0.446)
Environment	199	0.65	0.11	0.60	0.05	(0.503,0.703)

Note. N_Units is the number of sections included in each analysis. Inclusions is not a total, but the total number of sections assessed for eligibility. All other categories include sections which both raters agreed to include in the study. Pa is the percentage agreement, pe is the percent chance agreement, coeff.val is the AC1 agreement coefficient estimate, coeff.se is the standard error and conf.int is the AC1 confidence interval. All calculations were unweighted.

Table S2. Gwet's AC1 results for SW intra-rater reliability

Group	Units	pa	pe	coeff.val	coeff.se	conf.int
Inclusions	334	0.96	0.14	0.95	0.01	(0.93,0.98)
Individual dog	18	1.00	0.44	1.00	0.00	(1,1)
Dog population	18	0.72	0.49	0.46	0.22	(-0.002,0.921)
Dog owner	18	0.94	0.46	0.90	0.10	(0.676,1)
Human population	18	1.00	0.40	1.00	0.00	(1,1)
Environment	18	0.94	0.05	0.94	0.06	(0.81,1)

Note. N_Units is the number of sections included in each analysis. Inclusions is not a total, but the total number of sections assessed for eligibility. All other categories include sections which both raters agreed to include in the study. Pa is the percentage agreement, pe is the percent chance agreement, coeff.val is the AC1 agreement coefficient estimate, coeff.se is the standard error and conf.int is the AC1 confidence interval. All calculations were unweighted.

Table S3. Gwet's AC1 results for LMcD intra-rater reliability

Variable	n_Units	pa	pe	coeff.val	coeff.se	conf.int
Inclusions	121	0.85	0.35	0.77	0.06	(0.663,0.882)
Individual dog	18	0.89	0.22	0.86	0.10	(0.647,1)
Dog population	18	0.94	0.42	0.90	0.10	(0.695,1)
Dog owner	18	0.78	0.26	0.70	0.14	(0.403,0.994)
Human population	18	0.89	0.21	0.86	0.10	(0.651,1)
Environment	18	0.94	0.38	0.91	0.09	(0.717,1)

Note. N_Units is the number of sections included in each analysis. Inclusions is not a total, but the total number of sections assessed for eligibility. All other categories include sections which both raters agreed to include in the study. Pa is the percentage agreement, pe is the percent chance agreement, coeff.val is the AC1 agreement coefficient estimate, coeff.se is the standard error and conf.int is the AC1 confidence interval. All calculations were unweighted.

Pilot Study Results

In Pilot 1, we coded 142 sections from 11 laws and agreed to include 18. In Pilot 2, we coded 146 sections from 10 laws and agreed to include 11 sections. Percentage agreement and Cohen's Kappa were calculated for the law area and benefits from the sections we agreed to include.

Table S4. Pilot percentage agreement results

Variable	Pilot 1 pa	Pilot 2 pa	Percentage change
Inclusions	73%	84%	16%
Law Area	33%	55%	25%
Individual dog	33%	82%	51%
Dog population	33%	64%	13%
Dog owner	28%	64%	54%
Human population	28%	45%	39%
Environment	67%	73%	12%

Note. Pa is the percentage agreement and percentage change is the change from Pilot 1 to Pilot 2.

Table S5. Pilot 2 percentage agreement and Cohen's Kappa results

Variable	Pilot 1	Interpretation	Pilot 2	Interpretation	Change
Inclusions	0.319	Fair	0.479	Moderate	Improvement
Law Area	0.17	Slight	0.307	Fair	Improvement
Individual dog	0.0722	Slight	0.814	Almost Perfect	Improvement
Dog population	0.1	Slight	0.353	Fair	Improvement
Dog owner	-0.0588	Poor	0.476	Moderate	Improvement
Human population	0.069	Slight	0.0678	Slight	No change
Environment	0.4	Fair	0	Slight	Declined

Note. Values are Cohen's Kappa coefficient.
