# Supplementary material for: The Creation of a Systematic Framework to Assess Dog Laws and Their Relationship to Societal Changes in the United Kingdom
Source: Animals (Basel). 2025 Feb 23;15(5):647. doi: 10.3390/ani15050647 (PMC11898190; doi:10.3390/ani15050647)
Supplement: Supplementary file 1 [file animals-15-00647-s001.zip › File S4. Additional results.pdf]

File S4. Additional Results

Table S1. Summary of Benefits for Each Group Across Consolidated Legal Areas

Group	Benefited	Managing Dogs in Public	Dog Protections	Economic Activity	Transporting Dogs Across Borders
Individual Dog	Yes	46 (27%)	87 (81%)	32 (76%)	3 (38%)
	No	87 (51%)	10 (9%)	1 (2%)	5 (63%)
	No Impact	36 (21%)	10 (9%)	9 (21%)	0 (0%)
Dog Owner	Yes	42 (25%)	47 (44%)	35 (83%)	2 (25%)
	No	127 (75%)	60 (56%)	7 (17%)	6 (75%)
	No Impact	0 (0%)	0 (0%)	0 (0%)	0 (0%)
Human Population	Yes	139 (82%)	24 (22%)	5 (12%)	7 (88%)
	No	5 (3%)	15 (14%)	2 (5%)	0 (0%)
	No Impact	25 (15%)	68 (64%)	35 (83%)	1 (13%)
Dog Population	Yes	77 (46%)	72 (67%)	24 (57%)	6 (75%)
	No	19 (11%)	1 (1%)	0 (0%)	0 (0%)
	No Impact	73 (43%)	34 (32%)	18 (43%)	2 (25%)
Environment	Yes	21 (12%)	11 (10%)	0 (0%)	7 (88%)
	No	10 (6%)	0 (0%)	0 (0%)	0 (0%)
	No Impact	138 (82%)	96 (90%)	42 (100%)	1 (13%)
**Total sections in each consolidated law area**		**169**	**107**	**42**	**8**

[1.] Percentages were calculated using the total sections for each consolidated area and not all included sections.

Table S2. Comparison of stakeholder groups' benefits across nations

		Benefits		
Stakeholder Group	Nation	Yes	No	No Impact
Individual Dog	Northern Ireland	42% (45)	34% (37)	24% (26)
	Scotland	45% (46)	34% (35)	21% (21)
	England	42% (45)	39% (42)	19% (21)
	Wales	44% (50)	37% (42)	19% (21)
Dog Owner	Northern Ireland	38% (41)	62% (67)	0% (0)
	Scotland	40% (41)	60% (61)	0% (0)
	England	37% (40)	63% (68)	0% (0)
	Wales	38% (43)	62% (70)	0% (0)
Human Population	Northern Ireland	56% (61)	6% (6)	38% (41)
	Scotland	54% (55)	9% (9)	37% (38)
	England	53% (57)	6% (7)	41% (44)
	Wales	50% (57)	6% (7)	43% (49)
Dog Population	Northern Ireland	52% (56)	6% (7)	42% (45)
	Scotland	55% (56)	8% (8)	37% (38)
	England	47% (51)	7% (8)	45% (49)
	Wales	49% (55)	7% (8)	44% (50)
Environment	Northern Ireland	15% (16)	3% (3)	82% (89)
	Scotland	10% (10)	5% (5)	85% (87)
	England	15% (16)	5% (5)	81% (87)
	Wales	14% (16)	4% (5)	81% (92)

Table S3. Comparison of benefits afforded to stakeholder groups for Managing Dogs in Public across nations

Stakeholder Group	Nation	Benefited	Disadvantaged	Not Impacted
Individual Dog	Northern Ireland	21% (12)	53% (30)	26% (15)
	Scotland	27% (16)	58% (34)	15% (9)
	England	28% (19)	54% (37)	18% (12)
	Wales	28% (19)	54% (37)	18% (12)
Dog Owner	Northern Ireland	23% (13)	77% (44)	0% (0)
	Scotland	27% (16)	73% (43)	0% (0)
	England	25% (17)	75% (51)	0% (0)
	Wales	24% (16)	76% (52)	0% (0)
Human Population	Northern Ireland	86% (49)	4% (2)	11% (6)
	Scotland	83% (49)	3% (2)	14% (8)
	England	76% (52)	3% (2)	21% (14)
	Wales	76% (52)	3% (2)	21% (14)
Dog Population	Northern Ireland	46% (26)	11% (6)	44% (25)
	Scotland	51% (30)	14% (8)	36% (21)
	England	43% (29)	12% (8)	46% (31)
	Wales	44% (30)	12% (8)	44% (30)
Environment	Northern Ireland	11% (6)	5% (3)	84% (48)
	Scotland	12% (7)	8% (5)	80% (47)
	England	16% (11)	7% (5)	76% (52)
	Wales	16% (11)	7% (5)	76% (52)

Table S4. Comparison of benefits afforded to stakeholder groups for Dog Protections across nations

Stakeholder Group	Nation	Yes	No	No Impact
Individual Dog	Northern Ireland	84% (31)	11% (4)	5% (2)
	Scotland	83% (30)	3% (1)	14% (5)
	England	80% (28)	14% (5)	6% (2)
	Wales	83% (33)	13% (5)	5% (2)
Dog Owner	Northern Ireland	49% (18)	51% (19)	0% (0)
	Scotland	47% (17)	53% (19)	0% (0)
	England	43% (15)	57% (20)	0% (0)
	Wales	45% (18)	55% (22)	0% (0)
Human Population	Northern Ireland	19% (7)	11% (4)	70% (26)
	Scotland	22% (8)	19% (7)	58% (21)
	England	26% (9)	14% (5)	60% (21)
	Wales	23% (9)	13% (5)	65% (26)
Dog Population	Northern Ireland	70% (26)	3% (1)	27% (10)
	Scotland	72% (26)	0% (0)	28% (10)
	England	66% (23)	0% (0)	34% (12)
	Wales	68% (27)	0% (0)	33% (13)
Environment	Northern Ireland	14% (5)	0% (0)	86% (32)
	Scotland	8% (3)	0% (0)	92% (33)
	England	14% (5)	0% (0)	86% (30)
	Wales	13% (5)	0% (0)	88% (35)

Table S5. Comparison of benefits afforded to stakeholder groups for Economic Activity across nations

Stakeholder Group	Nation	Yes	No	No Impact
Individual Dog	Northern Ireland	57% (13)	4% (1)	39% (9)
	Scotland	60% (12)	0% (0)	40% (8)
	England	60% (12)	0% (0)	40% (8)
	Wales	67% (16)	0% (0)	33% (8)
Dog Owner	Northern Ireland	91% (21)	9% (2)	0% (0)
	Scotland	90% (18)	10% (2)	0% (0)
	England	90% (18)	10% (2)	0% (0)
	Wales	92% (22)	8% (2)	0% (0)
Human Population	Northern Ireland	9% (2)	0% (0)	91% (21)
	Scotland	5% (1)	5% (1)	90% (18)
	England	5% (1)	5% (1)	90% (18)
	Wales	4% (1)	4% (1)	92% (22)
Dog Population	Northern Ireland	35% (8)	0% (0)	65% (15)
	Scotland	55% (11)	0% (0)	45% (9)
	England	55% (11)	0% (0)	45% (9)
	Wales	54% (13)	0% (0)	46% (11)
Environment	Northern Ireland	0% (0)	0% (0)	100% (23)
	Scotland	0% (0)	0% (0)	100% (20)
	England	0% (0)	0% (0)	100% (20)
	Wales	0% (0)	0% (0)	100% (24)

Table S6. Comparison of benefits afforded to stakeholder groups for Transporting Dogs Across Borders across nations

Stakeholder Group	Nation	Yes	No	No Impact
Individual Dog	Northern Ireland	38% (3)	63% (5)	0% (0)
	Scotland	0% (0)	0% (0)	0% (0)
	England	0% (0)	0% (0)	0% (0)
	Wales	0% (0)	0% (0)	0% (0)
Dog Owner	Northern Ireland	25% (2)	75% (6)	0% (0)
	Scotland	0% (0)	0% (0)	0% (0)
	England	0% (0)	0% (0)	0% (0)
	Wales	0% (0)	0% (0)	0% (0)
Human Population	Northern Ireland	88% (7)	0% (0)	13% (1)
	Scotland	0% (0)	0% (0)	0% (0)
	England	0% (0)	0% (0)	0% (0)
	Wales	0% (0)	0% (0)	0% (0)
Dog Population	Northern Ireland	75% (6)	0% (0)	25% (2)
	Scotland	0% (0)	0% (0)	0% (0)
	England	0% (0)	0% (0)	0% (0)
	Wales	0% (0)	0% (0)	0% (0)
Environment	Northern Ireland	88% (7)	0% (0)	13% (1)
	Scotland	0% (0)	0% (0)	0% (0)
	England	0% (0)	0% (0)	0% (0)
	Wales	0% (0)	0% (0)	0% (0)
